# A Genetic Approach in the Evaluation of Short Stature

**DOI:** 10.5152/eurasianjmed.2022.22171

**Published:** 2022-12-01

**Authors:** Ayberk Turkyilmaz, Ayse Sena Donmez, Atilla Cayir

**Affiliations:** 1 Department of Medical Genetics, Karadeniz Technical University Faculty of Medicine, Trabzon, Turkey; 2 Department of Pediatrics, Regional Training and Research Hospital, Erzurum, Turkey; 3 Department of Pediatric Endocrinology, Regional Training and Research Hospital, Erzurum, Turkey

**Keywords:** Genetics, growth disorders/genetics, genetic predisposition to disease

## Abstract

Short stature is considered a condition in which the height is 2 standard deviations below the mean height of a given age, sex, and population group. Human height is a polygenic and heterogeneous characteristic, and its heritability is reported to be approximately 80%. More than 600 variants associated with human growth were detected in the genome-wide association studies. Rare and common variants concurrently affect human height. The rare variations that play a role in human height determination and have a strong impact on protein functions lead to monogenic short stature phenotypes, which are a highly heterogeneous group. With rapidly developing technologies in the last decade, molecular genetic tests have begun to be used widely in clinical genetics, and thus, the genetic etiology of several rare diseases has been elucidated. Identifying the genetic etiology underlying idiopathic short stature which represents phenotypically heterogeneous group of diseases ranging from isolated short stature to severe and syndromic short stature has promoted the understanding of the genetic regulation of growth plate and longitudinal bone growth. In cases of short stature, definite molecular diagnosis based on genetic evaluation enables the patient and family to receive genetic counseling on the natural course of the disease, prognosis, genetic basis, and recurrence risk. The determination of the genetic etiology in growth disorders is essential for the development of novel targeted therapies and crucial in the development of mutation-specific treatments in the future.

**Main Points**

- In short stature, the determination of genetic epidemiology plays an important role in the follow-up and treatment of other system pathologies.
- Molecular genetic studies guide the identification of rare short-stature variants.

- Molecular diagnosis in the short stature allows the family to receive genetic counseling.

## Introduction

Short stature (SS) is considered a condition in which the height is 2 standard deviations (SD) below the mean height of a given age, sex, and population group.^1^ Short stature is one of the most common causes of admission to pediatric endocrinology clinics. In addition to the constitutional delay of growth and puberty and familial short stature (FSS) known as “normal growth variants,” chronic diseases, hormonal diseases, and genetic causes play a role in the etiology of this condition.^1^ Idiopathic short stature (ISS) is a term used for children with SS without any systemic, endocrine, nutritional, or chromosomal abnormality. Idiopathic short stature comprises a wide range of patient group with phenotypic and genotypic heterogeneity, and most of the short children are followed up with this diagnosis.^1^ A multidisciplinary approach is required for the diagnosis of ISS, and clarification of the molecular diagnosis can be a guide in terms of follow-up and treatment.^1^

Growth and height growth in children is a multifactorial condition characterized by both genetic and environmental factors.^2^ The prevalence of pathological SS in different populations ranges from 1.3% to 19.8%.^3^ In a study conducted on school children in South India, the prevalence of SS was found to be 2.86%.^4^ Although the frequency of SS among children and adolescents in Shanghai was 3.26%, it was 0.7% in a study evaluating 79 495 children in Utah.^5,6^ In a study conducted on school children in the United Kingdom, the frequency of SS was 1.3%.^7^ In different studies evaluating school children in Turkey, the frequency of SS was found to be between 6.4% and 10.2%.^8-10^

Human height is a polygenic and heterogeneous characteristic, and its heritability is reported to be approximately 80%.^11^ More than 600 variants associated with human growth were detected in the genome-wide association studies (GWAS).^12-14^ Rare and common variants concurrently affect human height. Height differences within the normal range are related to common variants (multiple polymorphisms), and these variants are known to be involved in growth plate functions.^15,16^ The rare variations that play a role in human height determination have a strong impact on protein functions and lead to monogenic SS phenotypes, which are a highly heterogeneous group.^14^

With rapidly developing technologies in the last decade, molecular genetic tests have begun to be used widely in clinical genetics, and thus, the genetic etiology of several rare diseases has been elucidated.^17^ In diseases with high genetic heterogeneity such as ISS, microarray, methylation studies, and next-generation sequencing (NGS) technologies allow the determination of etiology in some cases. In a study on pediatric patients with isolated growth hormone deficiency and ISS, NGS was shown to be beneficial in determining the genetic etiology.^18^ In recent years, the evaluation of SS cases along with clinical findings and genetic analysis results has provided a better understanding of the clinical variability and genetic heterogeneity of SS syndromes.^19^ In cases of SS, definite molecular diagnosis based on genetic evaluation enables the patient and family to receive genetic counseling on the natural course of the disease, prognosis, genetic basis, and recurrence risk. In this study, we aimed to summarize the molecular mechanisms underlying the genetic causes of SS cases and to discuss the genetic approach algorithm to these cases.

## Molecular Genetic Mechanisms of Short Stature

### Defects in Hormonal Signaling Pathway (Growth Hormone/Insulin-Like Growth Factor 1 System)

Mutation in genes involved in the GH/IGF1 signaling pathway causes growth retardation and SS. Isolated GH deficiency is observed owing to defects in growth hormone 1 (*GH1*), growth hormone-releasing hormone receptor genes, and transcription factors (*HESX1, SOX2, SOX3, LHX3, LHX4, PTX1, PTX2, OTX2, PROP1, *and *POUF1*) involved in pituitary gland development in this pathway.^20^ Defects in GH receptor (*GHR*) and signal transducer and activator of transcription 5b (*STAT5B*) gene result in the development of GH resistance.^21,22^ In addition to SS, immune dysregulation is observed in *STAT5B* defects.^23^ In *IGF1* and *IGF1R* defects, intrauterine growth retardation (IUGR), microcephaly, and developmental delay are observed because these molecules also play a role in intrauterine development (Table 1).^24-26^

### Defects in Paracrine Signaling

Paracrine factors are effective in the proliferation and differentiation of chondrocytes in the growth plate.^27^ Paracrine signaling pathways include fibroblast growth factor (FGF)–FGF receptor signaling, parathyroid hormone-related protein and Indian hedgehog signaling, bone morphogenetic protein signaling, WNT signaling pathway, C-type natriuretic peptide signaling, and insulin-like growth factor 2 signaling. *FGFR3* mutations cause skeletal dysplasia phenotypes such as achondroplasia, hypochondroplasia, and thanatophoric dysplasia.^28-30^ Mutations in *PTHLH* and *PTH1R* cause brachydactyly type E2 and various types of skeletal dysplasia (Blomstrand lethal chondrodysplasia, Eiken syndrome, and metaphyseal chondrodysplasia Murk Jansen type), respectively.^31-33^ *IHH* mutations lead to brachydactyly type A1 and acrocapitofemoral dysplasia phenotypes.^34^ Although bone morphogenetic protein signaling defects cause brachydactyly, WNT signaling pathway *(ROR2*,* WNT5A*, and *DVL1*) anomalies cause Robinow syndrome.^35-37^ Biallelic inactivating mutations in the natriuretic peptide receptor 2 (*NPR2*) gene cause acromesomelic dysplasia Maroteaux type, and monoallelic mutations lead to the ISS phenotype.^38^ Paternal point mutations in *IGF2* and the loss of methylation at the imprinting control region (ICR1) site on chromosome 11p15.5 cause Russell–Silver syndrome (RSS) (Table 1).^38-40^

### Defects in Cartilage Extracellular Matrix

The extracellular matrix synthesized by chondrocytes comprising collagen, proteoglycan, and non-collagen proteins is vital for the structure and functions of the growth plate.^41^ Defects of genes involved in collagen synthesis (*COL2A1*, *COL9A1*, *COL9A2*, *COL9A3*, *COL10A1*, *COL11A1*, and *COL11A2*) cause different types of skeletal dysplasia.^42-45^ Defects of genes involved in the synthesis of non-collagen matrix proteins (*ACAN*, *COMP*, *MATN3*, *FBN1*, and *HSPG2*) cause SS, connective tissue anomalies, joint problems, and osteopenia (Table 1).^46-50^

### Defects in Constitutive Cellular Processes

Because the genes involved in constitutive cellular processes are crucial not only in the growth plate but also in all cells, findings such as microcephaly, skeletal dysplasia, and facial dysmorphism are observed in addition to SS and growth retardation in these cases. Defects in constitutive cellular processes can be divided into 3 groups according to its molecular mechanism.

#### Transcription Factors

Mutations in genes encoding transcription factors lead to various syndromic SS phenotypes. *SOX9* mutations cause campomelic dysplasia.^51^ Biallelic mutations of SS homeobox-containing gene (*SHOX*) lead to Langer mesomelic dysplasia, which is a severe skeletal dysplasia, whereas monoallelic mutations lead to Léri–Weill dyschondrosteosis phenotype, a milder skeletal dysplasia.^52^ Syndromic SS is noted in defects of several genes involved in transcription regulation (*LARP7*, *ANKRD11*,* CREBBP*,* EP300*,* KMT2D*, and *KDM6A)* (Table 1).^53-56^

#### DNA Repair

DNA repair defects cause severe SS, microcephaly, photosensitivity, leukemia, and syndromic SS that predispose to other types of cancer. Mutations in *ATR* and *ATR-ATRIP* complex, *RBBP8*, *DNA2*, and *TRAIP* cause Seckel syndrome, whereas mutations in *NBN *lead to progressive microcephaly, IUGR, increased sensitivity to ionizing radiation, and Nijmegen breakage syndrome, which causes premature ovarian failure in women.^57-61^ Mutations in *SMARCAL1*,* LIG4*, and* XRCC4* lead to Schimke immuno-osseous dysplasia, LIG4 syndrome, and “Short stature, microcephaly, and endocrine dysfunction” phenotypes, respectively (Table 1).^62-64^

#### Intracellular Signaling

Intracellular defects cause an extremely heterogeneous group of diseases that affect different signaling pathways, of which the most commonly known is the rat sarcoma (RAS)–mitogen-activated protein kinase pathway.^65^ Diseases that occur as a result of mutations in the genes involved in this signaling pathway are known as RASopathies.^66^ The most common RASopathy is Noonan syndrome, most commonly caused by mutations in *PTPN11* (50%).^67^ Other genes associated with this syndrome are *SOS1* (11%),* RAF1* (5%), *KRAS*, *NRAS*, *SHOC2*, and *BRAF*. LEOPARD syndrome, Costello syndrome, cardiofaciocutaneous syndrome, neurofibromatosis type 1, and Legius syndrome are among the other RASopathies group diseases. Common clinical findings of this group of diseases include SS, congenital cardiac anomalies, skin findings, neurocognitive retardation, characteristic facial findings, and cancer predisposition. Apart from this signaling pathway, *FGD1*, *GNAS1*, and *PI3K* mutations act on different intracellular pathways, leading to Aarskog–Scott syndrome, Albright hereditary osteodystrophy, and SHORT syndrome, respectively (Table 1).^68-70^

### Diagnostic Approach

The first step in the diagnostic examination of SS comprises obtaining family and patient history; physical examination; evaluation of growth velocity status and bone age; and a set of laboratory examinations. In pediatric endocrinology clinics, patients are evaluated in terms of FSS and constitutional delay in growth and puberty which are the variants of normal as well as hormonal dysfunctions and chronic diseases affecting growth as the first step of clinical examination. After hormonal examinations, targeted single gene analyses can be planned in cases with suspected anomalies in the GH/IGF1 signaling pathway. Careful evaluation of body proportion is important in the diagnosis of skeletal dysplasia, and in cases suspected of skeletal dysplasia, the entire skeleton should be evaluated with a bone survey.^71^ Skeletal dysplasia findings are detected in 22% of ISS cases, and this rate increases to 33% in the presence of an affected parent.^71^ In the evaluation of bone age, delayed or advanced bone age status can be detected in different clinical entities.^1,72,73^ The prevalence of *SHOX* deficiency in ISS cases varies between 3% and 15%.^74-77^ Therefore, the presence of radiographic findings (radiolucency, pyramidalization, and triangularization) on the wrist radiograph suggests that *SHOX* deficiency should be carefully evaluated.^78^ In addition, it should be noted that radiographic findings become evident in the late childhood period. In cases where SS is accompanied by dysmorphic findings, genetic consultation should be considered. Even if the typical stigmata of Turner syndrome are not found in girls with SS, karyotype should be requested.^79^

**Figure 1.**
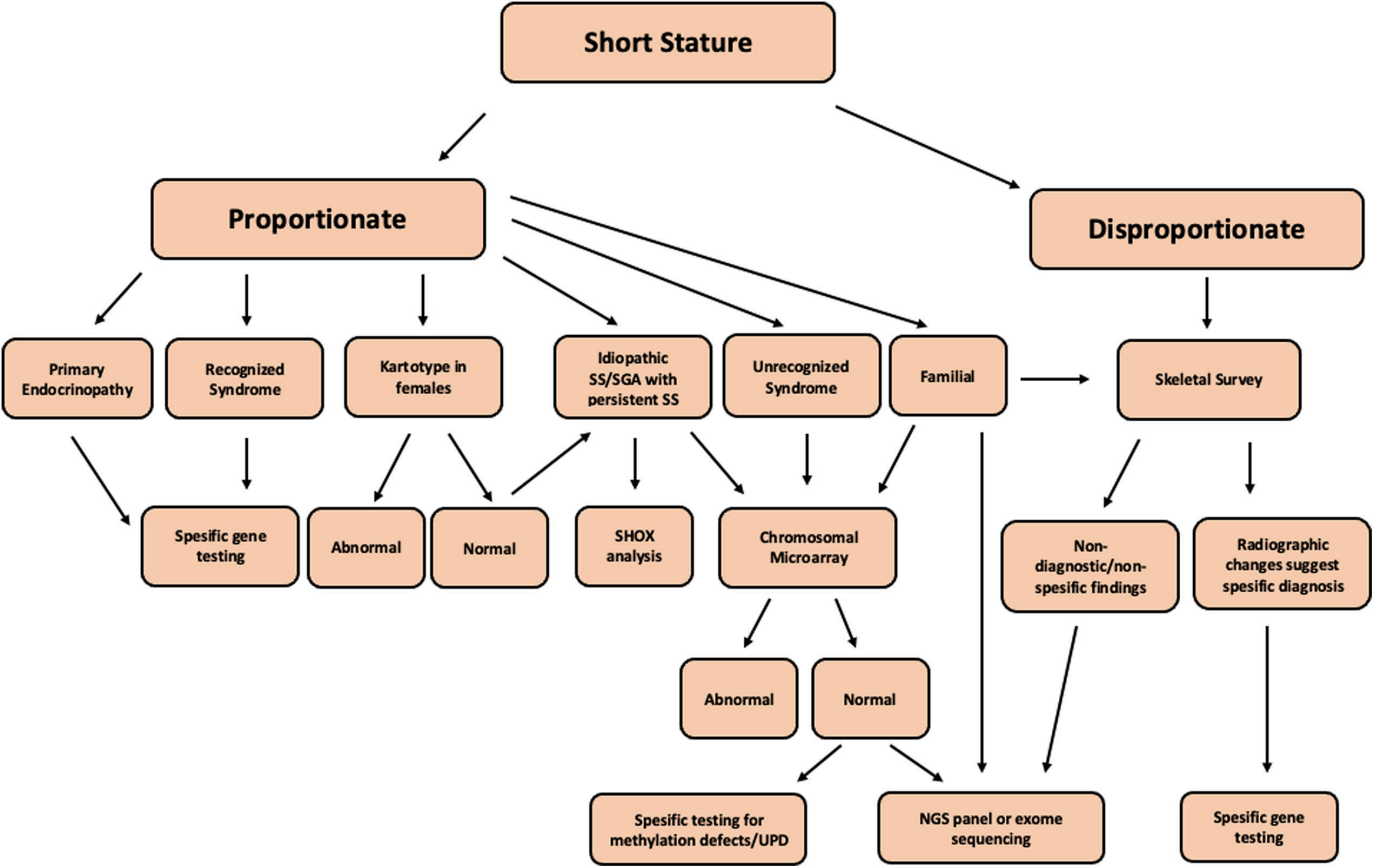
Algorithm for the genetic evaluation of short stature (this algorithm is proposed by American College of Medical Genetics and Genomics practice resource: Genetic evaluation of short stature).

“American College of Medical Genetics and Genomics practice resource": The algorithm suggested in the genetic approach to short stature cases according to the “Genetic evaluation of short stature” guideline is summarized in Figure 1.^80^ It is recommended to evaluate body proportion first. It is recommended to consider skeletal dysplasia in the first place in disproportionate cases and to request targeted analyses in the presence of specific diagnosis in radiographic evaluation. In the absence of specific diagnosis, it is recommended to evaluate using NGS panel or exome sequencing.^81^ Although targeted analyses are recommended in the presence of primary endocrinopathy or a recognizable syndrome in proportionate cases, karyotype is recommended in all female cases. In ISS cases with persistent SS, *SHOX* gene analysis is recommended if clinical findings are compatible and chromosomal microarray analysis (CGH and/or SNP) is recommended in the absence of a specific diagnosis. In cases with normal chromosomal microarray analysis, when methylation defect-related diseases (RSS, Temple Syndrome, etc.) are considered, analyses for methylation defects and uniparental disomy should be planned; alternatively, NGS panels or exome sequencing can be planned. Simultaneous or sequential chromosomal microarray analysis and NGS panel or exome sequencing are recommended for genetic etiology when FSS is suspected in proportionate cases.^80^ In this guideline, some special cases are emphasized. These conditions are as follows: (i) microarray analysis is recommended in females with persistent or evolving SS in which Turner syndrome is excluded. (ii) In cases of ISS, small for gestational age (SGA) with persistent SS, and syndromic short stature, it is recommended that chromosomal microarray analysis be applied as the first step in genetic test. The rate of diagnosis using chromosomal microarray analysis in this group of patients is 10%-15%.^81-85^ (iii) It is recommended to refer to the medical genetics departments for cases in which a specific diagnosis cannot be made but for which exome sequencing evaluation is required for suspected monogenic etiology. Particularly in cases with significant SS (height < −3 SD), facial dysmorphism, skeletal abnormalities, intellectual disability, microcephaly, multiple pituitary hormone deficiency, severe GH deficiency, SGA with persistent SS, family history of consanguinity, or family history of 1 parent with height of< −2SD are recommended to be consulted for further genetic analysis.^80^

### Genetic Testing Strategies

Using the genetic analyses mentioned in the previous section at appropriate time and order specific to patient will increase the rate of diagnosis. Genetic analyses used in the evaluation of SS cases can be performed through a wide range of conventional karyotyping, methylation analysis, microarray analysis, single-gene sequencing, NGS panel analysis, and exome sequencing. It should be noted that each technique has its advantages and limitations.

#### Chromosome Analysis

The first step in the evaluation of a child with SS is karyotyping. It is recommended for all female cases with SS suspected of having Turner syndrome or even without typical stigmata. Fluorescence in situ hybridization (FISH) analysis is used for diagnosis when microdeletion/microduplication syndromes that cannot be detected by karyotyping are suspected. Since 80%-90% of *SHOX *deficiency cases are associated with deletions, FISH analysis should be performed for the *SHOX* gene as the first step. In cases where deletion is not detected (10%-20% of the affected individuals), sequence analysis of the *SHOX* gene should be performed as the second step.^86^ It should be noted that FISH analysis may not be sufficient in some cases depending on the location and size of the deletion, and chromosomal microarray/multiplex ligation-dependent probe amplification analyses should be planned in these cases.^86^

Karyotyping and FISH analyses are used in the analysis of recurrent microdeletion/microduplication syndromes accompanied by SS. In addition to SS, microcephaly, facial dysmorphism, developmental delay, and congenital malformations can be seen in these syndromes.

#### Single Gene Testing

In ISS cases, targeted single-gene analyses should be selected if a specific single-gene defect is suspected based on the findings of clinical, laboratory, and radiographic examinations. Analyses of genes such as *GHR*, *IGF1*, *IGF1R*, *IGFALS*, and *PAPPA2* can be planned after clinical and laboratory evaluation of individuals with suspected GH/IGF-I axis defect. In cases where *SHOX* deficiency is considered, the test algorithm specified in the previous section should be applied. Although the clinical severity varies, sequence analysis for *ACAN* should be planned to investigate biallelic mutations in cases with spondyloepimetaphyseal dysplasia and severe SS.^72^ Mild signs such as skeletal dysplasia, SS, midface hypoplasia, and advanced bone age suggest monoallelic mutations in the ACAN gene.^72^ Heterozygous mutations in the ACAN gene were detected in 1.4% of ISS cases.^87^ Genetic analysis should be performed for the point mutation in *FGFR3* at c.1138G>A (p.Gly380Arg), which is known to be responsible for 99% of the cases, in patients with suspected achondroplasia. In cases with hypochondroplasia, point mutation analysis of *FGFR3* at c.1620C>A/G (p.Asn540Lys) noted in 70%-80% of cases should be performed. If mutation cannot be detected, whole gene sequencing of *FGFR3* should be requested. The genetic evaluation for suspected thanatophoric dysplasia type II and thanatophoric dysplasia type I include analysis of the point mutation in *FGFR3* gene at p.Lys650Glu which is known to cause 99% of the cases and at p.Arg248Cys & p.Tyr373Cys which are known to cause 90% of cases, respectively. If mutation cannot be detected, whole gene sequencing of *FGFR3 *should be requested. Although the prevalence varies in different studies, heterozygous loss-of-function mutations in the NPR2 gene have been detected in ISS cases and it has been shown that biallelic mutations cause acromesomelic dysplasia (Maroteaux type).^88^

#### Multi-Gene Testing Approach

In the absence of specific diagnosis in ISS cases, targeted NGS panels can be employed.^89-91^ Although the diagnosis rates with targeted NGS panels in ISS cases vary according to the gene content of the panel and patient selection criteria, it ranges between 2% and 8.7%.^89-91^ The most common diagnoses in cohorts evaluated with targeted NGS panels comprise Noonan syndrome (*PTPN11, SOS1*), statures, and advanced bone age, with or without early onset osteoarthritis and/or osteochondritis dissecans (*ACAN*), Stickler syndrome (*COL2A1*), and pseudoachondroplasia (*COMP*).^89-91^

#### Genome-Wide Testing Approach

Comparative genomic hybridization (Array-CGH) or SNP arrays platforms, which assess the whole genome for copy number variations (CNVs), and whole-exome sequencing (WES) analyses for the whole protein-encoding genome are comprehensive genetic tests that can be used in ISS cases. Comparative genomic hybridization (Array-CGH) or SNP arrays platforms are recommended to be applied before WES in ISS cases because they are cheaper and easily accessible.^80^

In the Array-CGH or SNP array studies conducted on ISS cases, CNVs’ detection rates were found to be approximately 10%-15%.^81-85^ Recurrent microdeletion/duplication syndromes were detected in some of the diagnosed cases, and the remaining had deletions/duplications containing genes associated with short stature.^81-85^

In cases with ISS with normal karyotype, microarray, and targeted NGS panel results, it has been reported that the diagnosis rates are between 16.5% and 46% with WES analysis.^92-95^ Analysis of all previously identified SS genes is possible through WES analysis. In addition, WES analysis is a genetic tool that enables the discovery of novel genes and signaling pathways associated with SS and growth retardation. Molecular diagnosis is easier in the presence of pathogenic or likely pathogenic variant compatible with the patient’s clinic; however, several variants of uncertain significance (VUS) are observed during WES analysis. During the interpretation of these VUS variants, all clinical, laboratory, and radiographic data of the patient and segregation analysis studies are used.

It should be noted that rare genetic syndromes that occur with epigenetic mechanisms may be responsible for the etiology of ISS cases.^96^ Russell–Silver syndrome is characterized by prenatal and postnatal growth retardation, feeding difficulties, recurrent hypoglycemia, body asymmetry, prominent forehead, and relative macrocephaly at birth.^97^ Molecular etiology can be detected in 60% of the cases, and the most common loss of methylation (30%-60%) on chromosome 11p15, followed by maternal uniparental disomy for chromosome 7 (5%-10%), is observed.^97^ In cases with suspected RSS, the first step in genetic evaluation should be methylation analysis for the chromosome 11p15 region and maternal UPD analysis for chromosome 7.^97^ In cases with normal test results, further analyses should be performed to investigate rarer causes (UPD16, UPD20 and *CDKN1C,* and *IGF2 *gene sequence analyses).^97^

The rate of diagnosis has increased in cases of ISS owing to genome-wide genetic analysis methods that have developed and become widespread in the last decade. Genetic analyses suitable for specific prediagnosis are planned after the identification of phenotypic findings in syndromic and non-syndromic SS. In recent years, the widespread use of genetic tests has led to the early diagnosis of cases with the reverse genetics (inverse genotype-first strategy) approach and contributes to the better identification of phenotypic findings of SSsyndromes.^98^

## Conclusion and Future Perspectives

Advances in the field of molecular genetics have made a serious contribution to the determination of the genetic etiology of ISS cases. Identifying the genetic etiology underlying ISS which represents phenotypically heterogeneous group of diseases ranging from isolated short stature to severe and syndromic SS has promoted the understanding of the genetic regulation of growth plate and longitudinal bone growth. The approach to cases of SS first comprises physical examination, family history, measurement of body proportion, definition of facial dysmorphic findings, analysis of laboratory and radiological findings, and evaluation of bone age. In the presence of specific prediagnosis, genetic evaluation methods should be planned in accordance with the genetic test algorithm recommended in the previous sections. Clarification of genetic etiology is helpful to obtain individualized medical management of patients, determine the prognosis, provide appropriate genetic counseling, and avoid unnecessary tests. The determination of the genetic etiology in growth disorders is essential for the development of novel targeted therapies and crucial in the development of mutation-specific treatments in the future.

## Figures and Tables

**Table 1.** Overview of Short Stature Syndromes

Molecular Genetic Mechanisms of Short Stature	Associated Genes	Clinical Phenotypes
Defects in hormonal signaling pathway	*GH1, GHRHR, HESX1, SOX2, SOX3, LHX3, LHX4, PTX1, PTX2, OTX2, PROP1, POU1F1, GHR, STAT5B, IGF1, IGF1R*	Isolated GH deficiency, Laron syndrome, GH resistance with immune dysregulation, IGF1 deficiency, IGF1 resistance (OMIM:#262400, 612781, 173100, 262650, 618157, 182230, 206900, 312000, 221750, 610125, 613986, 610125, 262600, 613038, 262500, 604271, 618985, 245590, 608747, 270450)
Defects in paracrine signaling	*FGFR3, PTHLH, PTH1R, IHH, ROR2, WNT5A, DVL1, NPR2, IGF2*	Achondroplasia, hypochondroplasia, thanatophoric dysplasia, brachydactyly type E2, Chondrodysplasia Blomstrand type, Eiken syndrome, Metaphyseal chondrodysplasia Murk Jansen type, Brachydactyly type A1, Acrocapitofemoral dysplasia, Robinow syndrome, acromesomelic dysplasia Maroteaux type, Russell–Silver syndrome (OMIM:#100800, 146000, 187600, 187601, 613382, 215045, 600002, 125350, 156400, 112500, 113000, 608747, 616331, 602875, 615923, 616255, 616489)
Defects in cartilage extracellular matrix	*COL2A1, COL9A1, COL9A2, COL9A3, COL10A1, COL11A1, COL11A2, ACAN, COMP, MATN3, FBN1, HSPG2*	Stickler syndrome, Spondyloperipheral dysplasia, Spondyloepiphyseal dysplasia Stanescu type, SMED Strudwick type, SED congenital, Platyspondylic skeletal dysplasia Torrance type, Czech dysplasia, multiple epiphyseal dysplasia, Metaphyseal chondrodysplasia Schmid type, Fibrochondrogenesis, Spondyloepimetaphyseal dysplasia aggrecan type, Short stature and advanced bone age with or without early-onset osteoarthritis and/or osteochondritis dissecans, Pseudoachondroplasia, Spondyloepimetaphyseal dysplasia, Borochowitz-Cormier-Daire type, Geleophysic dysplasia 2, Acromicric dysplasia, Dyssegmental dysplasia, Silverman-Handmaker type (OMIM:#132450, 619248, 200610, 608805, 609162, 156550, 150600, 604864, 165800, 177170, 154700, 255800)
Transcription factors	*SOX9, SHOX, LARP7, ANKRD11, CREBBP, EP300, KMT2D, KDM6A*	*Campomelic dysplasia, Langer mesomelic dysplasia, Leri-Weill dyschondrosteosis, Alazami syndrome, KBG syndrome, Rubinstein-Taybi syndrome, Kabuki syndrome (*OMIM:# 114290, 249700, 127300, 615071, 148050, 180849, 613684, 147920, 300867)
DNA Repair	*ATR and ATR-ATRIP complex, RBBP8, DNA2, TRAIP, SMARCAL1, LIG4, XRCC4*	Seckel Syndrome, Schimke immunoosseous dysplasia, LIG4 syndrome, “Short stature, microcephaly, and endocrine dysfunction” *(*OMIM:# 210600, 606744, 615156, 242900, 606593, 616541)

GH, growth hormone; IGF1, insulin-like growth factor I; LIG4, ligase IV; SED, spondyloepiphyseal dysplasia; SMED, spondylometaepiphyseal dysplasia.
